# Unpacking the complexity of longitudinal movement and recruitment patterns of facultative amphidromous fish

**DOI:** 10.1038/s41598-022-06936-8

**Published:** 2022-02-24

**Authors:** Rodrigo Ramírez-Álvarez, Sergio Contreras, Aurélien Vivancos, Malcolm Reid, Ruby López-Rodríguez, Konrad Górski

**Affiliations:** 1 Facultad de Ciencias, Universidad Católica de la Santísima Concepción, Concepción, Chile; 2 Centro de Investigación en Biodiversidad y Ambientes Sustentables (CIBAS), Universidad Católica de La Santísima Concepción, Concepción, Chile; 3 Departamento de Sistemas Acuáticos, Facultad de Ciencias Ambientales y Centro EULA – Chile, Universidad de Concepción, Concepción, Chile; 4 Geology Department, University of Otago, Dunedin, New Zealand; 5 Instituto de Ciencias Marinas y Limnológicas, Facultad de Ciencias, Universidad Austral de Chile, Valdivia, Chile

**Keywords:** Animal migration, Behavioural ecology, Freshwater ecology

## Abstract

Longitudinal movement plays fundamental role in habitat colonization and population establishment of many riverine fish species. Movement patterns of amphidromous fish species at fine-scales that would allow characterizing the direction of movement and factors associated with the establishment of specific life-history strategies (resident or amphidromous) in rivers are still poorly understood. We assess fine-scale longitudinal movement variability patterns of facultative amphidromous fish species *Galaxias maculatus* in order to unfold its life-history variation and associated recruitment habitats. Specifically, we analyzed multi-elemental composition along core to edge transects in ear-bones (otoliths) of each fish using recursive partitions that divides the transect along signal discontinuities. Fine-scale movement assessment in five free-flowing river systems allowed us to identify movement direction and potential recruitment habitats. As such, resident recruitment of *G. maculatus* in freshwater (71%) and estuarine (24%) habitats was more frequent than amphidromous recruitment (5%), and was linked to availability of slow-flowing lotic or lentic habitats that produce or retain small-bodied prey consumed by their larvae. We postulate that life-history variation and successful recruitment of facultative amphidromous fish such as *G. maculatus* in river systems is driven by availability of suitable recruitment habitats and natural hydrologic connectivity that allows fish movement to these habitats.

## Introduction

Understanding of longitudinal movement patterns (movement along rivers) of migratory fish is fundamental to elucidate drivers of their life-history variation as well as recruitment and habitat colonisation^1,2^. Furthermore, knowledge of the variability of movement and the use of longitudinal gradients within river systems provides the basis for comprehension of the relative ecological contributions of specific areas of the riverine ecosystem (e.g., headwater lakes, floodplain water bodies)^3^. Riverine populations of migratory fish may exhibit variable characteristics and are often connected to some degree^4,5^. This degree of connection, modulated by hydraulic connectivity and movement barriers, plays a fundamental role in fish population establishment and persistence in river systems and can strongly influence the source-sink dynamics of recruits^6,7^.

Diadromy is a type of migration characterised by regular and predictable movements between freshwater and marine environments and is often associated with profound physiological changes^8,9^. Due to differences in habitats occupied by adults, juveniles and larvae as well as life-history stage during which migrations occur diadromous migrations may be defined as amphidromous, anadromous or catadromous^10^. Amphidromy is the most frequent among diadromous migratory strategies^11^ and was classically described as spawning in freshwater riverine habitats with subsequent larvae transport downstream and development in marine habitats, and juvenile migration back to freshwater habitats to complete growth to adults^12^. However, recent studies have shown that some populations of amphidromous species may lose migratory traits and develop resident populations^13,14^. These facultative amphidromous fish species are characterised by a complex pattern of migratory and resident populations that develop depending on recruitment habitat availability^13,15,16^. Indeed, there is substantial variation among amphidromous species in terms of the stage of the life cycle where movement takes place and the direction of migration, which is why some authors suggested that amphidromy may be considered as spatially extensive benthic-pelagic migration where pelagic habitats provide larval recruitment habitats^15^.

*Galaxias maculatus* (Jenyns, 1842), commonly in Chile referred to as puye, is a facultative amphidromous fish species widely distributed in the Southern Hemisphere. Puye is often abundant in river systems of central-southern Chile and Patagonia, where it plays important ecological functions and forms part of traditional fisheries resource for gourmet food of high market value^16,17^. It can be found in estuaries, river channels and lakes and may display a diversity of amphidromous and resident life-history strategies^18,19^. Some ecological aspects of puye that relate to this life-history diversity i.e., diet variation^20,21^, recruitment habitats^13,19,22^, phenotypic plasticity^23–25^, and genetic diversity and structuring^26–28^ have been previously evaluated. However, our understanding of the variation of movement of puye remains rudimentary as previous studies have approximated their migratory life-histories by indirect methods (e.g., isotopic niche variation)^16,20^ or focused on specific large-scale movements between marine and freshwater habitats^13,29^. Therefore, longitudinal movement patterns of movement of this facultative amphidromous species in river systems and their movement ranges at fine-scales that would allow characterising the direction of movement and factors associated with the establishment of specific life-history strategies (resident or amphidromous) in rivers are unknown.

Analyses of trace elements present in fish ear bones (otoliths) have been frequently used as a tool to describe fish movement patterns^30^. Otoliths are calcified structures in the inner ear of teleost fish that are used as balance as well as swimming speed and acceleration receptors^31^. There is history of use of otoliths as time-resolved record of physiological aspects linked to growth and the environment of fish. As otoliths grow throughout the life of fish from the egg stage to the capture of an individual, trace elements from the environment are incorporated into the calcium carbonate matrix^32,33^. Therefore, elemental composition along a transect from the primordium (core) to the edge of the otolith represents chemical signature of aquatic habitats experienced by fish throughout its life. Traditionally, visual interpretation of elemental transects through otolith sections has been performed to qualitatively assess variations in concentrations of elements (e.g., strontium, barium) to discriminate movement between habitats based on established thresholds^34–37^. However, this approach may be subjective because it does not quantitatively capture the nature of multivariate change in elemental data and restricts analysis potential^38^. An alternative is to use multi-elemental data to identify trace elements that contribute most to the variation of chemical signal that allows detection of fine-scale longitudinal movements^39,40^. Machine learning methods allow analyses of multidimensional data sets and identification of chronological groups associated with specific locations allowing detection of fine-scale movement changes and direction of within a river system^41^.

The objective of this study was to evaluate fine-scale movement patterns of facultative amphidromous puye within five river systems located in central-southern Chile and to discuss ecological implications in establishment of life-history strategies that characterise populations of this species. Considering previous studies reporting genetic differences in amphidromous and resident populations^27,28^, as well as a differentiation in the use of food resources^16^, we expected that (1) populations of puye will have different movement patterns depending on the river system evaluated, (2) river systems characterised by freshwater recruitment habitats (headwater lakes or floodplains) will be characterised by higher frequency of resident populations.

## Methods

### Study area, fish sampling and processing

**Figure 1:**
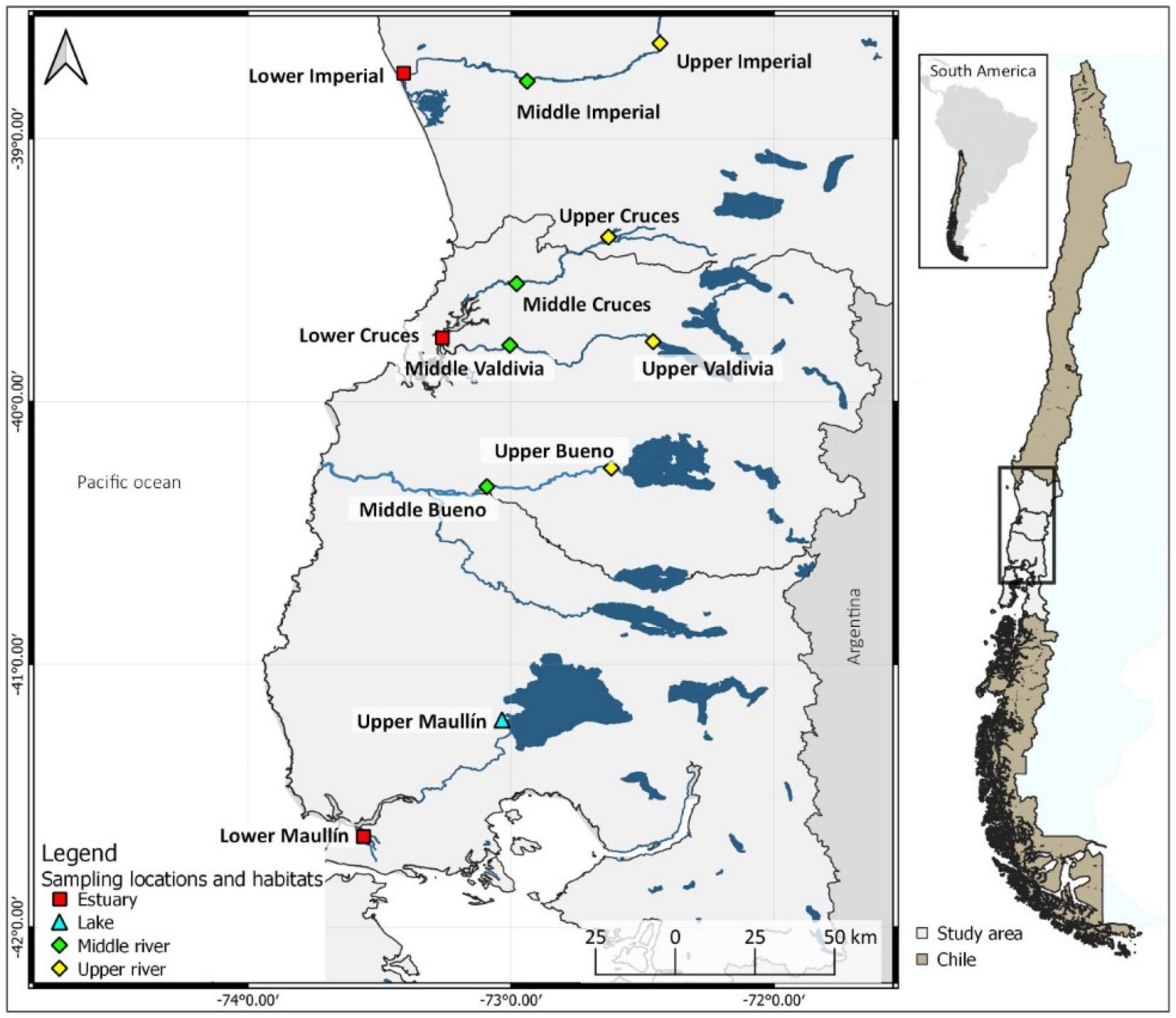
Location of puye *Galaxias maculatus* sampling sites in the Imperial, Cruces, Valdivia, Bueno and Maullín river systems. Map was created using QGIS version 3.16 (https://qgis.org/en/site/).

Fish were sampled in 12 locations in five river systems in central-southern Chile (Fig. 1) during March, May, August and November of 2019. Sampling sites were selected to cover a continuous river gradient including (whenever possible) lower, middle and upper section of each river system.

No artificial physical barriers that could obstruct fish movement were present between sampling sites. Fish were captured using beach seine (5 m long, 1.5 m high and 10 mm stretched mesh). In addition, physicochemical water parameters (temperature, pH, dissolved oxygen, conductivity, total dissolved solids) were measured using hand-held devices (Hanna HI-9828, Rhode Island, USA and Hanna HI-98703 Rhode Island, USA). At each sampling occasion, specimens collected were anesthetized, measured, weighed and identified in situ to species level. Subsamples of maximum 10 individuals of puye from each location in each sampling occasion (season) were frozen for further processing (depending on availability, and following guidelines of Chilean Undersecretariat for Fisheries and Aquaculture). In the laboratory, sagittal otoliths were extracted from each fish and any adhering tissue was cleaned away by rinsing with Milli-Q water. Subsequently, the left otolith of each individual was mounted sulcus side up onto glass microscope slides using CrystalBond™ 509 adhesive and sonicated for five minutes in Milli-Q water.

### Ethics statement

Capture methods and handling of animals were performed in accordance with institutional guidelines and regulations and were approved by the Ethics and Bioethics Committee of the Austral University of Chile (373/2019) and the Chilean Undersecretariat for Fisheries and Aquaculture (R. Ex No 4060/2017). The study was planned and conducted in accordance with ARRIVE guidelines^42^.

### Otolith elemental composition analyses

The elemental composition of each otolith was analysed using laser ablation inductively coupled mass spectrometry (LA-ICP-MS)^38,41,43^. We used an Agilent 7900 ICP-MS coupled to a Resonetics Resolution M-50 laser ablation system at the Trace Element Analysis Centre, University of Otago, Dunedin, New Zealand. Otolith mounts were arranged in the sampling cell and primordia were visually located using a 400× video imaging system. Each sample was repeatedly ablated in a vertical transect from the distal surface to the proximal surface through the primordium region (core) of each otolith, using a circular spot size of 75 µm, a repetition rate of 5 Hz an on-sample fluence of 2.5 J*cm^−2^ in pure helium environment. NIST 610 and 612 glass certified standards and MACS-3 matrix control (USGS) were run regularly to bracket groups of 10 samples throughout each laser session. The raw instrument intensity data were converted to elemental concentrations using the Trace Elements Data Reduction Scheme of Iolite software version 3.65^44^. The elements measured were Li (monitoring counts at m/z 7), B (11), Mg (25), Mn (55), Rb (85), Sr (88), Ba (183), K (39) and P (31). The internal standard was ^43^Ca with the concentrations expressed as molar ratios to calcium. NIST SRM 610 was used as calibration standard and NIST SRM 612 and MACS-3 as verification standards^45^. Elements for which > 25% of the measurements were below the detection limits were excluded from subsequent analyses.

### Assessment of chronological multi-elemental signatures

Variation in elemental composition along ablation transect of each otolith was identified by the chronological clustering method based on recursive partitions using the Tampo package in R^38^. This approach allows locating subtle chronological groups by detecting discontinuities in multivariate series, splitting otolith transects from the edge to the core. Because the signal structure is determined based on the signal chronology, this method was only possible in transects in which the otolith core was identified. We used manganese concentration peak in the signal to detect otolith cores as it was previously documented that otolith core region (primordium) is usually characterised by high concentration of manganese^46,47^. Considering the multi-elemental nature of the signal detected by chronological clustering, it is important to assess the relative contribution of the different elements in structuring the elemental signature (concentrations of detected elements along the transect or part of the transect; partition) that will allow selection of a subset of elements that best reflect the movement between sampling sites in each river system. This relative contribution was assessed using Random Forest models based on elemental signature of the otolith edges in each basin. The elemental signature recorded between 2 and 7 µm from the otolith surface was used as the edge signal since readings at distances < 2 µm were unreliable due to possible surface contamination and/or irregularities of the otolith surface. The most important elements that discriminated among sampling sites in each basin were those with the highest mean decrease in precision calculated using the *VarImpPlot ()* command in the R package *randomForest*.

Subsequently, to identify discontinuities in the elemental signature that can be associated with the transition between locations in each river system, unsupervised multivariate regression tree analyses were performed on subsets of elements previously selected by Random Forest analyses. Transect of each otolith was divided where elemental signature discontinuity was detected. First partition corresponded to the elemental signature recorded directly after the core region and the last partition corresponded to the elemental signature just before capture. Finally, the concentrations of elements in each partition were averaged to obtain their chemical fingerprint.

### Partition location prediction and movement history reconstruction

To predict the location of the different partitions of each otolith transect we followed methodology previously described^41^. First, Random Forest models were trained using independent measurement datasets for each river system. Subsequently these models were employed to make predictions about the location associated with the elemental signature of a given partition for a fish captured in a given section of the river. Random Forest models were trained with two data sets. First one included elemental signatures of the last partition of each otolith transect (the most homogeneous and recent multi-elemental signature of the otolith that likely reflected elemental composition of the last aquatic habitat experienced by the fish before it was captured^41^. For this dataset it is necessary to consider that not all fish have the same number of partitions. Since models were trained with last partitions, we have removed from the training data set those last partitions that will also be evaluated in the prediction in order to maintain a total independence between the training dataset and data used for location predictions. The second data set contained elemental signatures collected at the edges of transects in otolith for which it was not possible to identify the core region. For these otoliths, elemental signatures recorded between 2 and 7 µm from the otolith surface were used as the edge the signals (trace elements absorbed just before capture that were assumed to reflect elemental signatures of the capture sites).

Models developed for each river system were evaluated by determining Average Out-Of-Bag (OOB) classification accuracies based on 100 classification tests^48^. It was thus possible to quantify the ability of the models to correctly assign sample location from the elemental signatures of the training data set. The prediction of the location of a given partition was performed using the *predict()* function of the package *randomForest*, using the respective model. The location most voted by the model was associated with the evaluated partition, thus, all partitions of the transects were classified reconstructing the movement of each fish throughout its lifespan.

### Movement patterns and associated fish life-histories

To assess specific movement patterns between different habitats we first evaluated the differences in elemental signatures among estuarine and freshwater habitats. To do this, we compared multi-elemental compositions (Euclidean distance matrix) recorded at the edges of individuals captured in different habitats (estuary, lower river, upper river, lake) through permutational multivariate analysis of variance (PERMANOVA)^49,50^. We only used data sets from the last partitions (edges) in which we were certain of the type of chemical composition of the habitat. We considered the chemical signal of estuarine habitats as that recorded by individuals captured in estuaries and the chemical signal of freshwater habitats as that recorded by fish captured in the upper sections of each river system. For river systems where it was not possible to sample estuarine habitats (Valdivia and Bueno), the detection of estuarine elemental signatures was evaluated by training Random Forest models on data from all sampled estuaries (Imperial, Cruces, Maullín). Subsequently, the prediction of these elemental signatures in each partition was performed, to show if a given partition was associated with elemental signature of estuarine habitats. The detection of one or more partitions associated with estuarine elemental signature was interpreted as evidence in favour of amphidromous life-history.

## Results

Otolith elemental signatures of a total of 283 individuals of puye was evaluated. Clear manganese concentration peaks allowed identification of cores in 86 individuals. The number of individuals with core identified varied among sampling locations and ranged between 11 (Upper Maullín, Lower Valdivia) and 3 (Upper Cruces; Table 1). Multi-elemental analyses of 86 individuals revealed differences in elemental signatures among sampled locations.

**Table 1:** Number of individuals with cores captured in the river systems evaluated.

River system	Sampling location	N core identified	N core not identified
Bueno	Upper	7	17
Bueno	Middle	9	14
Cruces	Upper	3	21
Cruces	Middle	4	20
Cruces	Lower	5	19
Imperial	Upper	4	12
Imperial	Middle	4	20
Imperial	Lower	7	7
Maullín	Upper	11	13
Maullín	Lower	10	14
Valdivia	Upper	11	13
Valdivia	Middle	9	11

Otolith elemental signatures were predominantly structured by variation in concentration of strontium (^88^Sr), rubidium (^85^Rb), barium (^138^Ba), lithium (^7^Li), manganese (^55^Mn), boron (^11^B) and magnesium (^25^Mg) (Table S1). Other assessed elements i.e., phosphorus (^31^P) and potassium (^39^K) contributed less (mean decrease accuracy < 5% for all river systems) to the structure of elemental signatures and were excluded from subsequent analyses.

### Partition location predictions and reconstruction of fish movements

**Figure 2:**
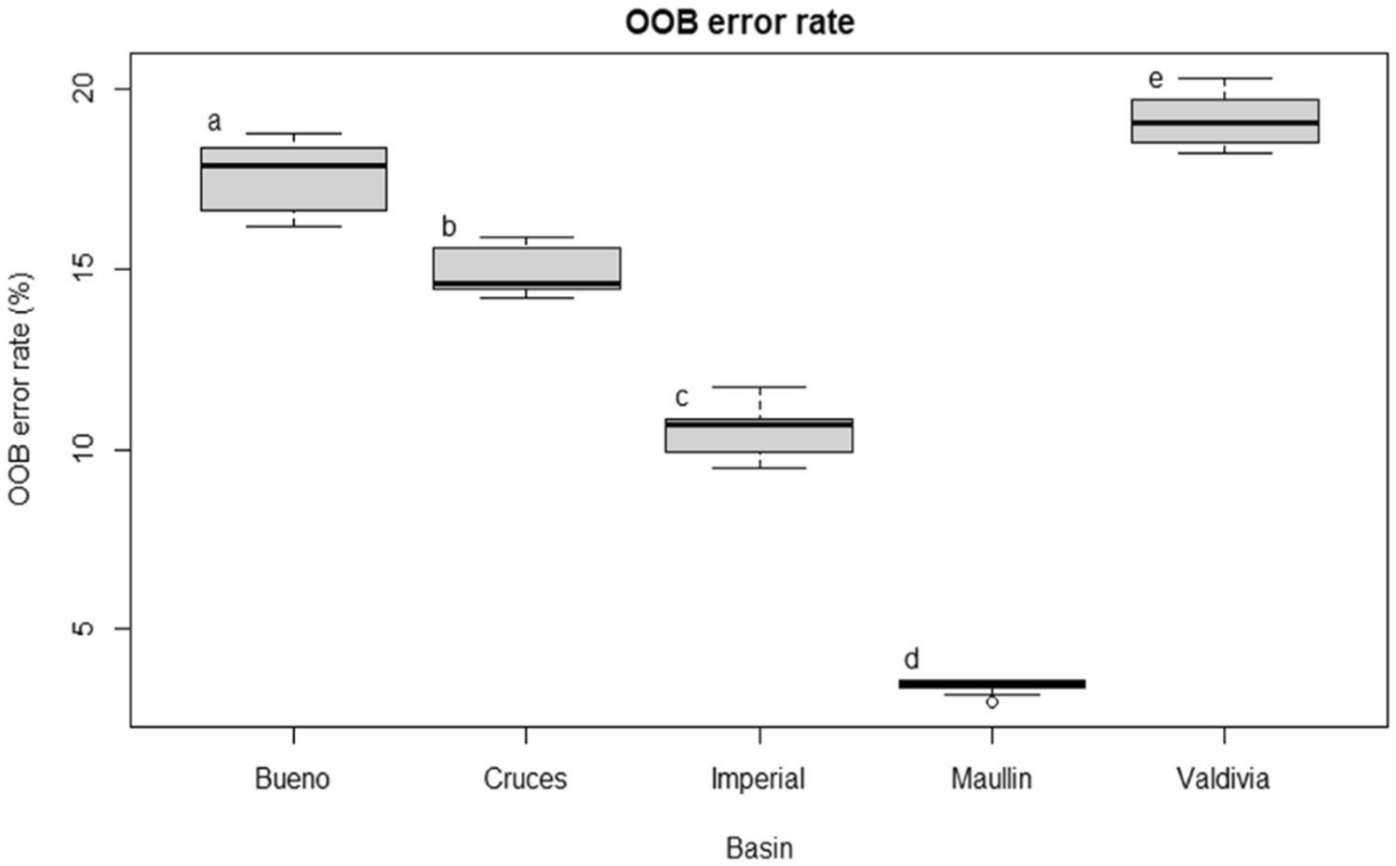
Out Of Bag (OOB) error rate (mean +/− s.d.) for the Random Forest models trained for each river system and each partition. Black lines indicate median values with surrounding upper and lower quartiles at the box edges. Mean and standard error were estimated from 100 simulations. Different letters indicate significant differences (ANOVA, F = 419.4, *p* < 0.001).

Random Forest models trained to predict the location of partitions reported low OOB error rates and classified capture location correctly from the otolith edge data set (prediction accuracy 80%). OOB was significantly different between rivers (*p* < 0.05), however, it was relatively constant among partitions from each location (Fig. 2). River systems with the highest variation among partitions were Bueno (18%) and Valdivia (15%) of the observed OOB. Maullín River was characterised by the lowest OOB error rate (3%) similar for all partitions.

**Figure 3:**
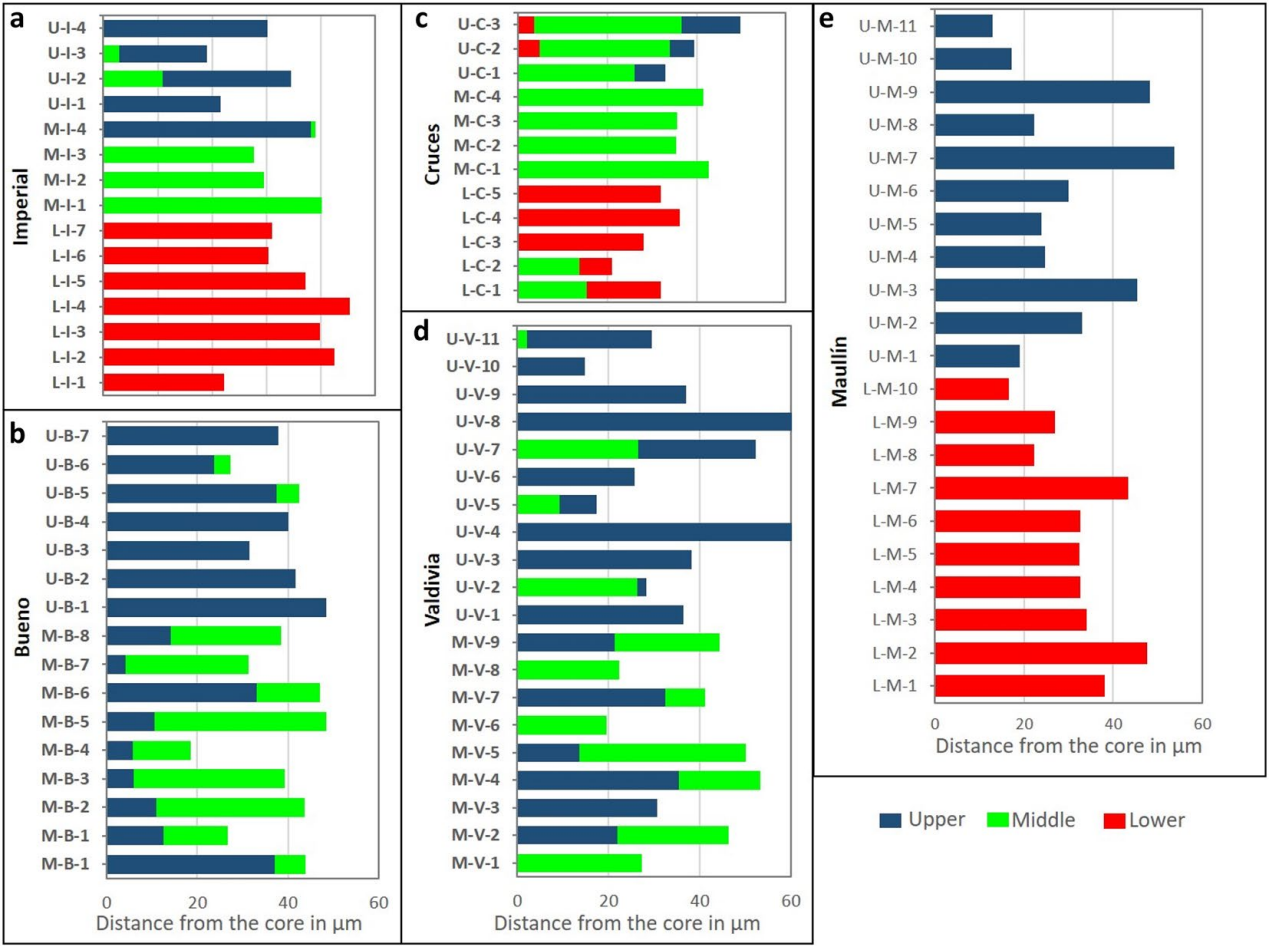
River section prediction for fish from each river systems (**a** Imperial River, **b** Bueno River, **c** Cruces River, **d** Valdivia River; **e** Maullín River). Each bar represent an otolith transect, going from the core (emergence) to the capture (death). Colour code matches the section of the river that has been predicted by the Random Forest model for each partition of the transect. Change of colour throughout the transect indicates a movement occurring during the lifespan of the fish.

We found significant variation of reconstructed movement history among analysed river systems (Fig. 3, Fig. S1). Otoliths elemental signatures of fish captured in the Imperial River system revealed a variety of movement histories. As such, all fish captured in the lower section (estuary) were characterised by homogeneous elemental signatures suggesting they have remained there throughout their lifespans. Furthermore, there were no fish caught in the upper and middle sections that were characterised by elemental signatures of the lower section suggesting no upstream movement of estuarine fish (Fig. 3a). Of the four fish captured in the middle section of the Imperial River, three remained in their capture site throughout their lifespans whereas one (M-I-4) remained most of its life in the upper section and moved to the middle section just before capture (Fig. 3a). This result was obtained with a high proportion of votes (> 90%). Two individuals captured in the upper section remained in their capture site throughout their lifespans (U-I-1 y U-I-4), whereas other two were characterised by elemental signatures of their first partitions associated with the middle section of the river suggesting upstream movement (U-I-2 y U-I-3).

Of the 16 fish captured in upper and middle sections of the Bueno River, 5 remained in their capture location throughout their lifespan and 11 were characterised by partitions associated to different locations indicating movement between sections (Fig. 3b). Interestingly, all fish captured in the middle section were characterised by first partitions associated with the upper section, suggesting natal origins upstream and subsequent movement downstream. For these fish, predictions were based on a high voting ratio (more than 80% of all the votes). Finally, two fish captured in the upper section were characterised by last partitions predicted to the middle section of the river.

In the Cruces River, five fish were characterised by heterogeneous elemental signatures (three in the upper section and two in the lower section) suggesting movement between sections, whereas four fish captured in the middle section and three in the lower section were characterised by homogeneous elemental signatures suggesting that they remained in the capture site throughout their lifespans (Fig. 3c). Elemental signatures revealed that two fish (U-C-2 y U-C-3) moved from the lower section (estuary) of the river through the middle to the upper section (this movement pattern was predicted with a high proportion of votes (> 80% for all three sections). Furthermore, two fish captured in the lower section (L-C-1 y L-C-2) were characterised by initial partitions associated with the middle section, indicating their movement downstream.

In the Valdivia River, nine fish were characterised by partitions associated with different localities, indicating movement of fish between river sections (Fig. 3d). Amongst these fish, four were captured upstream, but the first partitions were predicted for the middle section of the river, indicating upstream movement. In contrast, five fish capture in the middle section had their first partitions associated with the upper section, indicating downstream movement (> 80% of the votes). In the Maullín River, elemental signatures of all fish were homogeneous along transects predicting all partitions associated with capture location indicating no movement between the river sections evaluated (Fig. 3e). These predictions were based on high voting ratio (> 95%).

### Differences in chemical signatures between estuarine and freshwater habitats

Individuals captured in different habitats significantly differed in multi-elemental signatures of their otolith edges (PERMANOVA Pseudo-F: 218,03, *p* = 0.0001). Pair-wise comparisons indicated significant differences among all habitats with the exception of lake and upper river freshwater habitats (Table 2). This difference was consistent with significant differences in concentrations of individual elements among habitats (Supplementary Table S2). These differences were driven by higher strontium concentrations in estuaries and higher rubidium concentrations in freshwater habitats. Furthermore, concentrations of lithium, manganese also significantly differed among freshwater habitats (Supplementary Table S2).

**Table 2:** Post hoc pairwise comparisons (PERMANOVA) of multi-elemental signatures of *Galaxias maculatus* otolith edges among habitats. Asterisks indicate significant differences, P < 0.05).

Habitat	t	P(perm)
Lake, Upper river	0.97095	0.3753
Lake, Middle river	2.0892	0.0249*
Lake, Estuary	9.4838	0.0001*
Upper river, Middle River	2.0985	0.0314*
Upper river, Estuary	17.106	0.0001*
Middle river, Estuary	17.379	0.0001*

Location prediction of partitions among individuals captured in estuarine and freshwater habitats did not predict any of the partitions different from the expected elemental signatures of capture sites (vote proportion > 95%). Furthermore, fish from Bueno and Valdivia river systems (where no estuarine locations were sampled), did not report estuarine elemental signatures in any of their partitions.

### Movement patterns of amphidromous and resident populations

**Figure 4:**
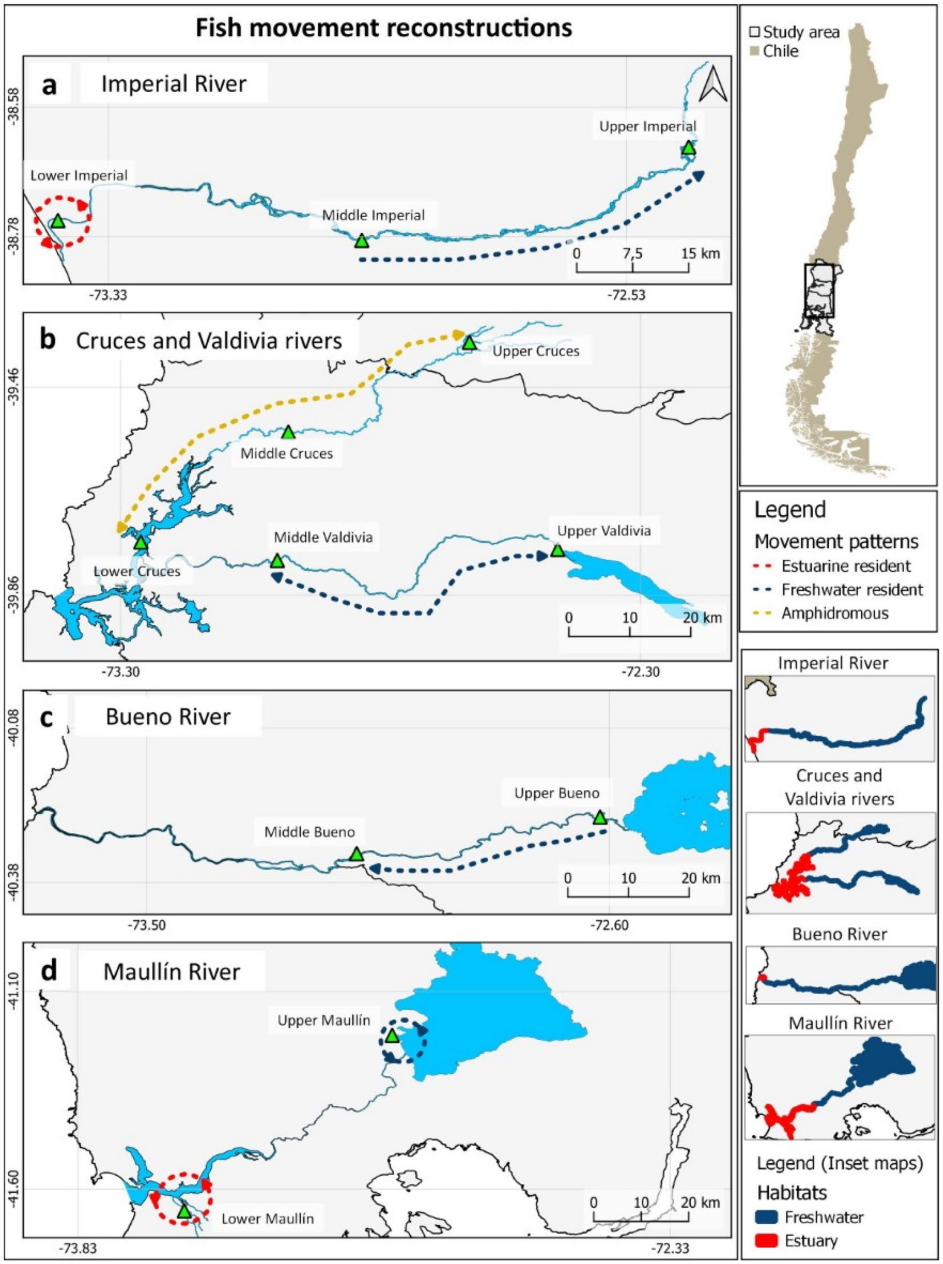
Reconstruction of movement patterns of *Galaxias maculatus* captured in 5 rivers in the central-southern Chile (**a** Imperial River, **b** Cruces and Valdivia rivers, **c** Bueno River, **d** Maullín River). The arrows indicate the predicted movement based on the partition location prediction analysis (Random Forest models). Colour of each arrow corresponds to life-history estimated for each river system: red, estuarine resident; blue, freshwater resident; yellow, amphidromous. Inset maps show the extent of estuarine and freshwater habitats. Map was created using QGIS version 3.16 (https://qgis.org/en/site/).

Based on the reconstruction of fish movement among sampling locations in relation to elemental signatures of estuarine and freshwater habitats, different life-history strategies can be identified across evaluated river systems (Fig. 4). Fish captured in the upper and middle sections of the Imperial River showed maximum distance of directional displacement of 79 km (equal to the distance between the two sites; Fig. 4a), but absence of estuarine elemental signatures along their lifespan suggest resident populations restricted to freshwater habitats (Fig. 4 Inset map). Furthermore, fish captured in the lower section of the Imperial River did not seem to move to freshwater habitats. Similar pattern was reported in the lower section of the Maullín River where movement reconstruction also indicated estuarine residency (Fig. 4d). Furthermore, fish captured in the upper section of Maullín (headwater lake) were characterised by freshwater resident life history based on elemental signatures in their otoliths (Fig. 4 Inset map).

Among river systems analysed, we detected the highest maximum displacement distances in fish captured in the Cruces River system (Fig. 4b). The predicted partitioning associated with the three locations indicates upstream movement of approximately 149 km from estuarine to freshwater habitats suggesting amphidromous life-histories (Fig. 4 Inset map). In contrast, maximum detected displacement of fish captured in the Valdivia River system was limited to the middle and upper sections of the river (approximately 72 km), without elemental signatures associated with estuaries, suggesting populations with freshwater resident life-history. Similarly, detected maximum displacement of fish from the Bueno River system was restricted to locations within freshwater habitats and the distance of approximately 64 km (Fig. 4c).

## Discussion

Analyses of multi-elemental concentrations of fish otoliths reported in this study allowed to elucidate within river movement patterns of riverine fish including specific direction of movement. As such, we could characterise natal origins and recruitment habitats that unpacked the scale of life-history variation strongly linked to suitable habitat availability in a river system. Indeed, we report high prevalence of resident recruitment of puye and for the first time establishment of resident populations in estuarine habitats.

Studies that characterise fish movement patterns based on otolith micro-chemical analyses have mainly been based on strontium and calcium concentration ratios^13,29,51^. This is mostly due to high discriminatory capacity of Strontium to detect movement between marine and freshwater environments^30,52^. However, some facultative amphidromous fish such as puye can be characterised by complex movement and migration strategies mediated by interplay of species’ reproductive aspects and specific ecological characteristics that condition movement between different habitat types^53,54^. Furthermore, although amphidromous migration (i.e., movement between marine and freshwater environments) may not be present, longitudinal movements among river sections within river system (upstream and downstream) remain significant features of fish ecology that are poorly understood especially for small bodied fish species^55,56^. Here, we used multivariate chronological clustering analysis to generate multi-elemental signatures of otolith edges, which were subsequently linked to chemical fingerprints of sampled localities. Robustness of our analyses, however, depends on the ability of the models to discriminate elemental signatures between localities. The evaluation of Random Forest models showed a low OOB error rate, assigning between 80 and 93% (depending on the river system) of the chemical signals from otolith edges to their capture sites. The predictive power of the models varied from location to location and reflected complex nature of the elemental signatures that strongly depends on the river system considered. However, all partitions were consistently predicted within each river system, suggesting that the multi-elemental signature was able to capture complex interactions of each river section within the river system. As such, our results corroborate complex nature of fish otoliths’ chemical signatures and that use of multiple elements provides higher discriminatory power compared to use of single element^38,40^. Indeed, several previous studies have described movement patterns of fish from changes in elemental ratios detected sequentially along a section of otoliths^29,36,57^, however, identifying movements at fine-scales, i.e., within river systems is a more complex task and it strongly depends on the capability to match the elemental signatures with fish capture locations. In this study, using machine learning methods, we were able to link otolith elemental signatures to characteristics of sampling locations and identify movement not only between estuarine and freshwater habitats, but also between sections of each river system, thus reconstructing fine-scale movements available for just few small bodied fish species^39,41^.

We quantitatively elucidated within river movement patterns of puye in each of the river system studied. In the Bueno and Valdivia rivers, a significant proportion of the fish captured reported continuous movement between the upper and middle sections of each river system, with maximum detected displacement of 64 km (Bueno River) and 72 km (Valdivia River). This movement pattern corroborates recent findings of similar resource use and significant overlap of isotopic niche spaces of puye in upper and middle sections of both Valdivia and Bueno rivers^20^. Predictive analyses did not associate any of the fish captured in Valdivia and Bueno river systems with estuarine elemental signatures suggesting restriction of their life-histories to freshwater habitats possibly distinct from estuarine populations. Similarly, no movement of puye was detected between estuaries and upper sections of Maullín and Imperial rivers. Indeed, elemental signatures of estuarine fish were consistently different form signatures of fish captured in upper sections. These differences were strongly driven by significantly higher concentrations of strontium in estuarine fish, and higher concentrations of rubidium in fish captured in freshwater (Supplementary Table S2). This result is consistent with previous studies that reported higher strontium concentrations in otoliths of fish captured in marine and estuarine habitats compared to those captured in fresh water^51,58–60^. Lack of movement between estuarine and freshwater habitats in these river systems may limit gene flow between populations inhabiting these habitats that would result in genomic differences that were recently reported between resident and estuarine populations in south-central Chilean rivers^27,28^. Cruces River was the only river system where high proportion of movement among all river sections and amphidromous life-histories were detected. Indeed, in this river system fish characterised by early life-history in estuarine habitats were captured in upper river section approximately 149 km from the estuary.

Our results provided evidence of amphidromous and freshwater resident populations, but also estuarine resident populations that were previously considered amphidromous^16,20,27,28^. Amphidromy was described as regular physiologically mediated movements between freshwater and marine habitats, however, we detected that the estuarine populations of the Imperial and Maullín rivers do not move towards freshwater environments and remain in estuaries during their entire lifespan^9^. Puye is a facultative amphidromous species and some previous studies have documented freshwater recruitment and establishment of freshwater resident populations^13,16,61^. To date, however, no estuarine resident populations have been reported. This finding is corroborated by Manosalva et al.^20^ who reported that isotopic niches of estuarine and freshwater populations of puye in the Imperial and Maullín rivers did not overlap.

We document variation of life-histories employed by populations of puye in Chilean river systems that is strongly linked to movement patterns, where most populations are resident. Establishment of resident populations can be driven by several factors such as availability of spawning and nursery habitats and connectivity among essential habitats within the river system that allow recruitment of juveniles^15,19,62^. Juvenile and adult puye inhabit littoral zones of rivers and lakes characterised by low current velocities where they feed on zooplankton and benthic invertebrates^17,20^. As such, successful reproduction and recruitment of puye in freshwater habitats depend on the availability of slow-flowing lotic or lentic habitats that retain larvae and provide small-bodied prey consumed by them^15,19,63,64^. The establishment of resident populations reported in the Bueno, Valdivia and Maullín rivers could be mediated by the contribution of large headwater lakes to the direct supply of zooplankton and other food resources that allow juvenile recruitment. Furthermore, our results contrast those of Górski et al.^16^ and report freshwater resident populations of puye also in the Imperial River. Indeed, this previous study evaluated resource use that does not permit assessment of movement direction, whereas here we present fine-scale movement analyses that allowed to elucidate movement direction and potential recruitment habitats. Imperial River does not originate in headwater lake and establishment of resident populations in this system could be influenced by the presence of floodplains or low gradient lateral riverine areas that provide larval nursery habitats and produce or retain zooplankton that subsidies larval food sources^65,66^. Indeed, topographic analysis of Imperial River allowed detection of gentle river slopes, open valleys and floodplain areas in the middle section of the river (Supplementary Fig. S2) that could explain the detected recruitment in the middle section of the river and subsequent upstream movement (Fig. 3a). In contrast, Cruces River system is characterised by extensive estuarine wetland in the lower reaches, but confined valleys in the upper reaches (Supplementary Fig. S2). Probably these geomorphological conditions have favoured amphidromous life-histories and limited establishment of resident populations. Indeed, all fish captured in the upper reaches of the Cruces River were characterised by natal origins in lower sections (Fig. 3c).

We should be aware that identification of fish movement by analyses of otolith elemental signatures is a complex task that requires integration of multivariate analytical tools and an understanding of otolith chemical responses to the interactive effects of environmental and endogenous variables^38,40,67^. Reconstructing the environmental history of fish from otolith chemistry is based on the assumption that during biomineralisation process the incorporation of elements will depend mainly on elements available in the water they inhabit. However, some authors suggest that the assimilation capacity of elements in otoliths may be influenced by the interaction of different environmental factors, diet, physiological processes, and even species-specific variation^57,68–70^. Our models were trained based on concentrations in otolith edges themselves and therefore elemental signatures we used for predictions were already influenced by many of these processes. Furthermore, the effects of abiotic and biotic factor on incorporation of chemical elements in the otoliths may vary among individuals and their developmental stages^69,71^. We used chemical signals in otolith edges of various individuals from each location in order to incorporate individual and ontogenic variation within the chemical fingerprint of each location. Moreover, elements that contributed to discrimination among habitats and locations of puye within river systems considered in our study i.e. strontium, rubidium, barium, boron, lithium were shown to be relatively reliable predictors of environmental histories of fish^72^. We used data collected in a sampling campaign between March and November 2019, therefore seasonal variability of water elemental composition could affect our results. We tested the effects of season in elemental signatures using PERMANOVA and found no significant differences among seasons for each location with exception of Upper Bueno (Supplementary Table S3). We concluded that the effect of season on water elemental concentrations is most probably less important than spatial effects that allow movement assessment based on elemental signatures^73^.

Several phylogeographic studies have proposed that diadromy is puye’s ancestral trait^27,74^, however, it is not an obligatory strategy. When suitable conditions for larval recruitment exist in specific locations such as floodplains or lakes connected to the river system, resident populations can establish. Fine-scale movement assessment in five free-flowing river systems (no physical barriers that disrupt hydraulic connectivity) allowed us to identify movement direction and potential recruitment habitats. We found significant variation in displayed life-histories, with dominance of resident populations in both freshwater and estuarine habitats that are linked to suitable habitat availability in a river system. This life-history variation results in high genetic diversity of puye^27^. Therefore, recruitment of puye populations is strongly linked to natural hydrologic connectivity and availability of specific nursery habitats. As such, anthropogenic activities that affect natural hydrologic connectivity are expected to significantly affect recruitment of puye populations.

## Supplementary Information

Supplementary Information.

## Data Availability

Data that support the findings of this study are available from the corresponding author upon reasonable request.
