# Supplementary material for: Unpacking the complexity of longitudinal movement and recruitment patterns of facultative amphidromous fish
Source: Sci Rep. 2022 Feb 24;12:3164. doi: 10.1038/s41598-022-06936-8 (PMC8873245; doi:10.1038/s41598-022-06936-8)
Supplement: Supplementary file 1 — Supplementary Information. [file 41598_2022_6936_MOESM1_ESM.docx]

**Supplementary Information.** Ramírez-Álvarez, R., S. Contreras, A. Vivancos, M. Reid, R. López-Rodríguez and K. Górski. 2022. Unpacking the complexity of longitudinal movement and recruitment patterns of facultative amphidromous fish.

Supplementary Table S1. Mean accuracy decrease (%) for each element in each river system.

|  | **Sr** | **Rb** | **Ba** | **Li** | **Mn** | **B** | **Mg** |
| --- | --- | --- | --- | --- | --- | --- | --- |
| Bueno | 13 | 15 | 11 | 14 | 11 | 11 | 3 |
| Cruces | 35 | 4 | 7 | 2 | 6 | 0 | 4 |
| Imperial | 25 | 16 | 5 | 17 | 4 | 2 | 0 |
| Maullín | 19 | 20 | 7 | 11 | 3.5 | 3.5 | 0 |
| Valdivia | 7 | 0 | 0 | 0 | 7 | 3 | 0 |

Supplementary Table S2. Comparison of concentrations (mmol/mol Ca) of individual elements from otolith edges among habitats. Asterisks indicate significant differences (**, p< 0.001; *, p<0.01)

|  | Lake (L) | | Upper river (UR) | | Middle river (MR) | | Lower river - Estuary (E) | | F | Tukey post-hoc test |
| --- | --- | --- | --- | --- | --- | --- | --- | --- | --- | --- |
|  | n=23 | | n=77 | | n=84 | | n=56 | |  |  |
|  | Mean | *sd* | Mean | *sd* | Mean | *sd* | Mean | *sd* |  |  |
| Sr | 6,19E-04 | *7,13E-05* | 6,39E-04 | *1,80E-04* | 7,02E-04 | *1,96E-04* | 3,13E-03 | *1,25E-03* | 223.5** | E > UR, MR, L |
| Mg | 1,00E-04 | *1,51E-04* | 6,66E-05 | *7,61E-05* | 5,27E-05 | *3,47E-05* | 9,15E-05 | *1,40E-04* | 2.69 |  |
| Ba | 4,09E-06 | *3,65E-06* | 2,98E-06 | *1,39E-06* | 3,34E-06 | *1,83E-06* | 3,01E-06 | *3,37E-06* | 1.49 |  |
| Rb | 8,69E-07 | *2,59E-07* | 1,06E-06 | *5,20E-07* | 9,12E-07 | *5,15E-07* | 3,19E-07 | *1,56E-07* | 33.1** | UR, MR, L > E |
| Li | 6,56E-07 | *5,27E-07* | 1,31E-06 | *1,18E-06* | 9,63E-07 | *1,06E-06* | 1,38E-06 | *1,22E-06* | 3.69* | E, MR > UR, L |
| B | 3,91E-05 | *9,20E-05* | 1,66E-05 | *8,73E-06* | 2,22E-05 | *1,58E-05* | 1,79E-05 | *8,52E-06* | 3.52* | E, MR, UR > L |
| Mn | 4,20E-06 | *2,00E-06* | 6,87E-06 | *4,24E-06* | 8,28E-06 | *6,35E-06* | 5,62E-06 | *5,94E-06* | 4.86* | MR > E, UR, L |

**Seasonal variation analyses**

We used data collected in a sampling campaigns during March (summer), May (autumn), August (winter) and November (spring) to assess seasonal variability of water elemental composition (approximately 6 individuals in each season and location, Table S2). We tested the effects of season in elemental signatures using PERMANOVA and found no significant differences among seasons for each location with exception of Upper Bueno (Table S2).

Supplementary Table S3. PERMANOVA of seasonal variation (summer, autumn, winter, spring) in elemental concentrations at each location. Asterisk indicates significant differences.

|  | Sampling locations (Sections) | Summer N | Autumn N | Winter N | Spring N | Total N | F | *P* |
| --- | --- | --- | --- | --- | --- | --- | --- | --- |
| Bueno | Upper | 6 | 6 | 6 | 6 | 24 | 4.68* | 0.0008* |
| Bueno | Middle | 6 | 5 | 6 | 6 | 23 | 1.43 | 0.23 |
| Cruces | Upper | 6 | 6 | 6 | 6 | 24 | 2.03 | 0.08 |
| Cruces | Middle | 6 | 6 | 6 | 6 | 24 | 1.11 | 0.36 |
| Cruces | Lower | 6 | 6 | 6 | 6 | 24 | 0.96 | 0.43 |
| Imperial | Upper | 5 | 3 | 4 | 4 | 16 | 0.29 | 0.78 |
| Imperial | Middle | 6 | 6 | 6 | 6 | 24 | 0.31 | 0.88 |
| Imperial | Lower | 2 | 3 | 4 | 5 | 14 | 3.77 | 0.06 |
| Maullín | Upper | 6 | 6 | 6 | 6 | 24 | 1.11 | 0.32 |
| Maullín | Lower | 6 | 6 | 6 | 6 | 24 | 0.10 | 0.98 |
| Valdivia | Upper | 6 | 6 | 6 | 6 | 24 | 0.81 | 0.60 |
| Valdivia | Middle | 5 | 6 | 4 | 5 | 20 | 1.85 | 0.11 |
| Season (Total) | | 66 | 65 | 66 | 68 |  |  |  |


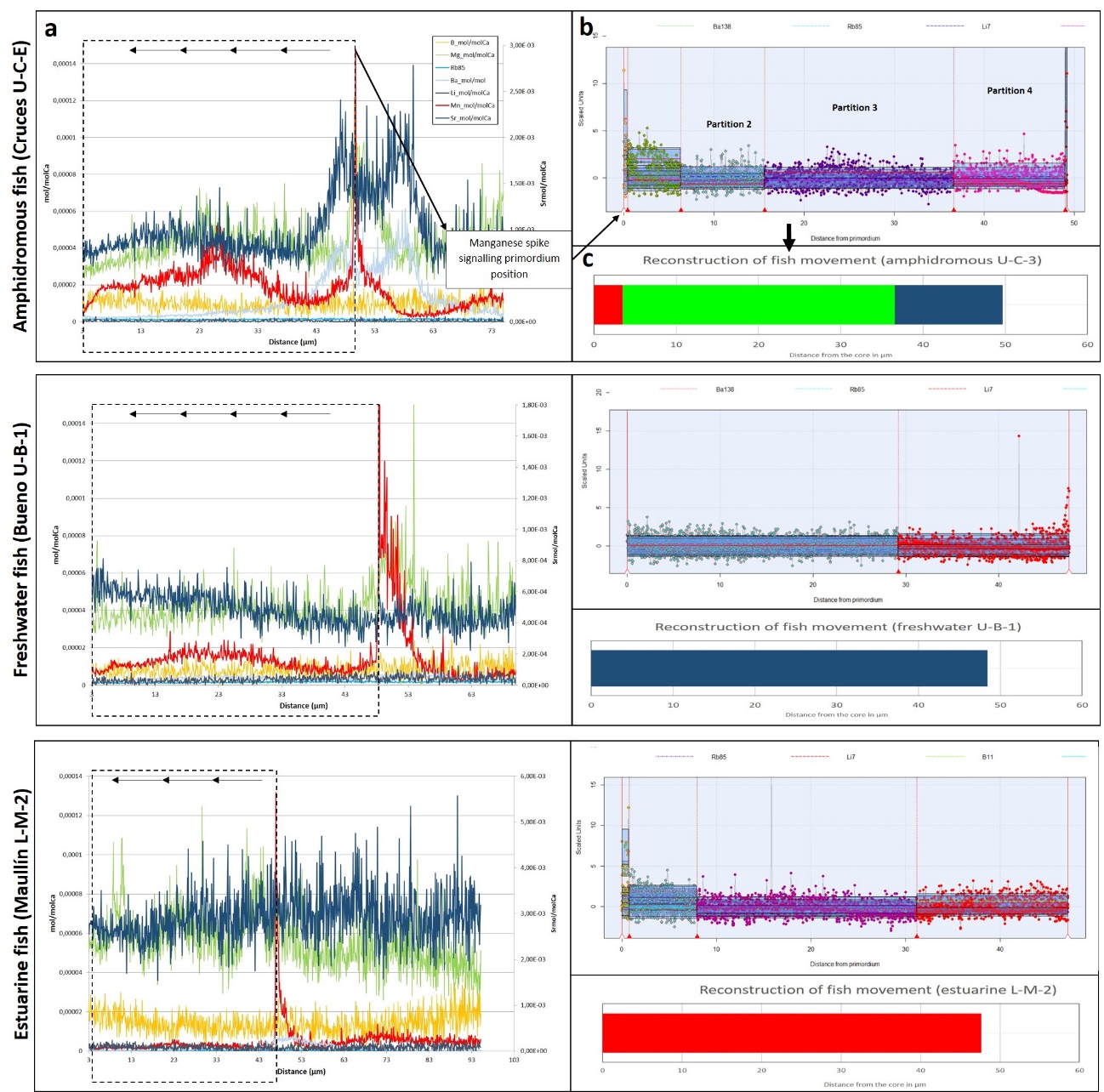


Supplementary Figure S1. Example of otolith transect analyses of three specimens of *Galaxias maculatus* corresponding to three different life histories observed (estuarine resident, freshwater residents and amphidromous). In this example, a spike of manganese was detected in the multi-elemental signal (a), which indicated that the transect went through the primordium (core) and could be used for movement reconstruction (dashed box and black arrows indicate data transect considered for the analyses). Chronological clustering was performed on multi-elemental signal from the primordium to the edge (b). Locations were then predicted using random forest models for each partition in order to identify movement among sampled sites (c).


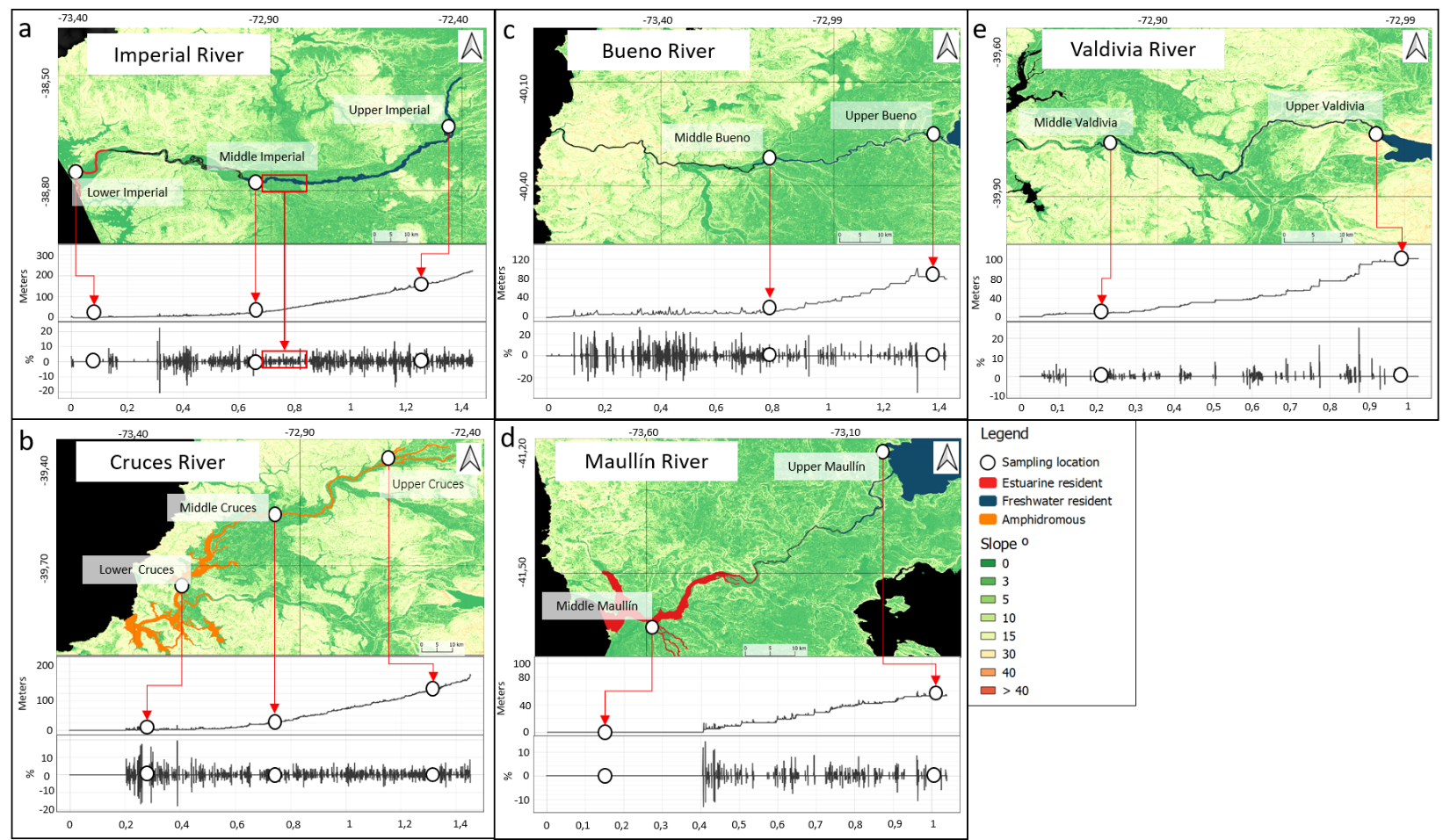


Supplementary Figure S2. Topographic analyses of studied river systems using a 30m digital terrain model: Imperial River (a), Cruces River (b), Bueno River (c), Maullín River (d), Valdivia River (e). Figure was created using QGIS version 3.16 (<https://qgis.org/en/site/>). The map shows the slope gradient represented in degrees. Elevation profile (calculated in meters) and slope profile (in %) of the main stem of each river system are given below each map. High frequency of high values in slope profile represent high slope variation in a river section. The red rectangle specified in the Imperial River (a) shows areas with low slope and floodplain habitat.
